# A retrospective study on nested PCR detection of syphilis treponemes in clinical samples: PCR detection contributes to the diagnosis of syphilis in patients with seronegative and serodiscrepant results

**DOI:** 10.1371/journal.pone.0237949

**Published:** 2020-08-20

**Authors:** Eliška Vrbová, Lenka Mikalová, Linda Grillová, Petra Pospíšilová, Radim Strnadel, Eliška Dastychová, Martina Kojanová, Miluše Kreidlová, Daniela Vaňousová, Filip Rob, Přemysl Procházka, Alena Krchňáková, Vladimír Vašků, Vladana Woznicová, Monika Dvořáková Heroldová, Ivana Kuklová, Hana Zákoucká, David Šmajs

**Affiliations:** 1 Department of Biology, Faculty of Medicine, Masaryk University, Brno, Czech Republic; 2 Department of Dermatovenerology, Faculty Hospital Brno, Brno, Czech Republic; 3 Department of Medical Microbiology, Faculty of Medicine, St. Anne’s Hospital and Masaryk University, Brno, Czech Republic; 4 Department of Dermatology, 1st Faculty of Medicine, Charles University in Prague, Prague, Czech Republic; 5 Institute of Medical Biochemistry and Laboratory Diagnostics of the General University Hospital and of The First Faculty of Medicine of Charles University in Prague, Prague, Czech Republic; 6 Department of Dermatovenerology, 2nd Faculty of Medicine, Charles University in Prague, Prague, Czech Republic; 7 Outpatient STI Clinic Medicentrum, Prague, Czech Republic; 8 National Reference Laboratory for Diagnostics of the Syphilis, National Institute for Public Health, Prague, Czech Republic; University of Lincoln, UNITED KINGDOM

## Abstract

Syphilis, caused by *Treponema pallidum* ssp. *pallidum* (TPA), is a persisting global health problem. Although syphilis diagnostics relies mainly on serology, serological tests have some limitations, and it is recommended that the final diagnosis be supported by additional tests. The purpose of this study was to analyze the relationship between serology and PCR in syphilis diagnostics. From the year 2004 to May 2019, a total of 941 samples were taken from 833 patients suspected of having syphilis, in Czech Republic. In all these samples, both nested PCR detection of TPA and serology testing were performed. Of the 941 samples, 126 were seronegative, 651 were seropositive, and 164 were serodiscrepant. Among seronegative samples (n = 126), 11 were PCR-positive (8.7%). Among seropositive samples (n = 651; i.e., samples positive for both non-treponemal and treponemal serology tests), 368 samples were PCR-positive (56.5%). The remaining 164 serodiscrepant samples included RPR negative and treponemal serological test-positive samples (n = 154) and a set of 10 RPR-positive samples negative in treponemal serological tests. While the first group revealed 73 PCR-positive samples (47.4%), the second revealed 5 PCR positive samples (50.0%). PCR detection rates were highest in primary syphilis, with lower rates in the secondary and undetermined syphilis stages. As shown here, the nested PCR can improve diagnostics of syphilis, especially in seronegative patients and in patients with discrepant serology.

## Introduction

Syphilis is a multi-stage venereal disease, which is caused by the *Treponema pallidum* subsp. *pallidum* (TPA) bacterium. Worldwide, there are over 5 million new cases of syphilis every year [1], and about 700 new cases are reported annually in the Czech Republic (Institute of Health Information and Statistics of the Czech Republic). In most cases, a syphilis diagnosis is based on clinical observations, anamnestic data, and results of serology testing. Serological tests are divided into two groups, non-treponemal and treponemal. To diagnosis syphilis, results from both types of tests are required. Non-treponemal tests include the Rapid Plasma Reagin (RPR) test and the Venereal Disease Research Laboratory (VDRL) test. These tests detect antibodies against cardiolipin, high titers of which are found in ongoing infections. Treponemal tests include the Fluorescent Treponemal Antibody Absorption Test (FTA-Abs), *T*. *pallidum* Hemagglutination Assay (TPHA), and *T*. *pallidum* Particle Agglutination Assay (TPPA). TPHA/TPPA detects, nonselectively, both IgM and IgG antibodies, while IgM and IgG can be detected separately using ELISA, FTA-Abs, and Western blot (WB). Whereas TPHA/TPPA and ELISA tests are often used for screening, FTA-Abs and WB tests are used for confirmation [2]. Although serological tests have, in general, a high specificity and sensitivity, they have several limitations: (1) there is reduced sensitivity in the early and late stages of syphilis, (2) there is a risk of false-positive reactions, which can be caused by other acute or chronic infections, and (3) the non-treponemal tests are susceptible to false-negative results due to the prozone effect [1, 3, 4]. Since 1990, direct detection of treponemal DNA based on PCR has been used more frequently, although, still not routinely [5, 6]. Previous studies have also shown that the best samples for treponemal DNA detection come from swabs taken from syphilitic ulcers, rather than whole blood samples [7, 8].

The major advantages of PCR TPA detection include the specificity of PCR, TPA detection in the very early stages of syphilis, and detection of TPA in patients with congenital syphilis and neurosyphilis [9]. In addition, PCR detection and subsequent sequencing allow molecular typing of the specific TPA circulating within a population, and differentiating among the various *T*. *pallidum* subspecies [10–12] for clarification of diagnosis.

Several previous studies, which compared PCR and serological testing, have reported different results. Brischetto et al. [13] considered serology to be sufficient for diagnosis without PCR, while others [14, 15] considered PCR to be complementary to serology since each technique has its own advantages as well as limitations.

In this communication, we compare the results of several serological methods, both non-treponemal tests (i.e., RPR) and treponemal tests (i.e., TPPA, ELISA, and WB analysis of IgM and IgG) with PCR detection of treponemal DNA from swab and whole blood samples. We also analyzed samples with discrepant serology results as well as seronegative samples.

## Material and methods

### Collection of clinical samples and syphilis serology

Clinical samples were collected from patients in the Czech Republic from 2004 to May 2019. In total, 941 clinical samples from 833 patients with suspected syphilis were collected. Four clinical departments (Department of Dermatovenerology, St. Anne´s Faculty Hospital; Department of Dermatovenerology, Faculty Hospital Brno, Masaryk University; Department of Dermatovenereology, 1st Faculty of Medicine, Charles University and the National Reference Laboratory for Diagnostics of Syphilis, National Institute for Public Health) were involved in the samples collection. Collected samples included swabs from skin ulcers (n = 8), oropharyngeal ulcers (n = 29), genitoanal ulcers (n = 54), ulcers with unspecified location (n = 402) and/or whole-blood samples (n = 448). Swab and blood sample was simultaneously collected from 108 patients. Whole blood samples were drawn into commercially available tubes, containing 5.4 mg of K_2_EDTA. Swabs were transported in a dry state in a sterile capped tube with either no liquid transport medium or in 500 μl of phosphate buffered saline (PBS). Samples from Brno were transported on ice to the laboratory, samples from Prague were transported on dry ice. Samples were stored at −80°C.

Serological testing was performed for all patients. Results of both nontreponemal and treponemal tests were provided by recruiting centers. Tests included the RPR test (IMMUTREP RPR, Omega Diagnostics Ltd, Alva, UK; RPR reditest, Biokit, Barcelona, Spain), TPPA test (TP-PA Serodia, FUJIRebio, Tokyo, Japan), ELISA/WB analyses of IgM (TestLine Clinical Diagnostics s.r.o., Brno, CZ; MarDx® Syphilis IgM Western Blot, Trinity Biotech, Bray, Ireland) and ELISA/WB analyses of IgG levels (TestLine Clinical Diagnostics s.r.o., Brno, CZ; MarDx® Syphilis IgG Western Blot, Trinity Biotech, Bray, Ireland). The syphilis stage was determined by clinicians using the CDC case definitions [16], i.e., clinical manifestation of primary syphilis characterized by one or more chancres (ulcers); secondary syphilis characterized by localized or diffuse mucocutaneous lesions, which could be accompanied with generalized lymphadenopathy.

### DNA isolation

DNA was isolated from 200 μl of clinical samples as described previously [17] using QIAamp DNA Blood Mini Kits (Qiagen, Hilden, Germany). Extraction was performed within 24 hours after samples being received.

### PCR detection

Samples were tested for the presence of treponemal DNA using an advanced nested PCR protocol (developed for TPA molecular typing) containing touchdown PCR and using PrimeSTAR GXL polymerase (Takara, BioEurope, France) to increase detection power of nested PCR [18–24]. PCR was performed for four to six loci, including TP0136, TP0548, TP0705, 23S rRNA genes, TP0319 (*tmpC*), and TP0105 (*polA*) as described previously [17–20, 25]. Details of PCR mixtures, protocols, and a list of primers are shown in S1 Table [18, 25].

### Statistical methods

Correlations between results from PCR and serology were tested using the two-sided Fisher´s exact test, and statistical significance was set at p < 0.05. Comparisons of RPR titer values were tested using the unpaired t-test after logarithmic transformation. Agreement between PCR and serology was assessed by calculation of the kappa coefficient. Sensitivity, specificity and predictive values, were determined individually for PCR and serology. Our case definition was based either on positive results of both types of serological tests (i.e., non-treponemal and treponemal) and/or on PCR positivity (defined as positive amplification of at least two loci). Statistical analyses were performed using STATISTICA software v.12 (StatSoft, Tulsa, OK, USA).

### Ethics statement

The study was approved by the ethics committee of the Faculty of Medicine, Masaryk University (5G/2017). All clinical samples were collected after the patient’s written informed consent.

## Results

### Serology and PCR results on the set of collected samples

**Fig 1:**
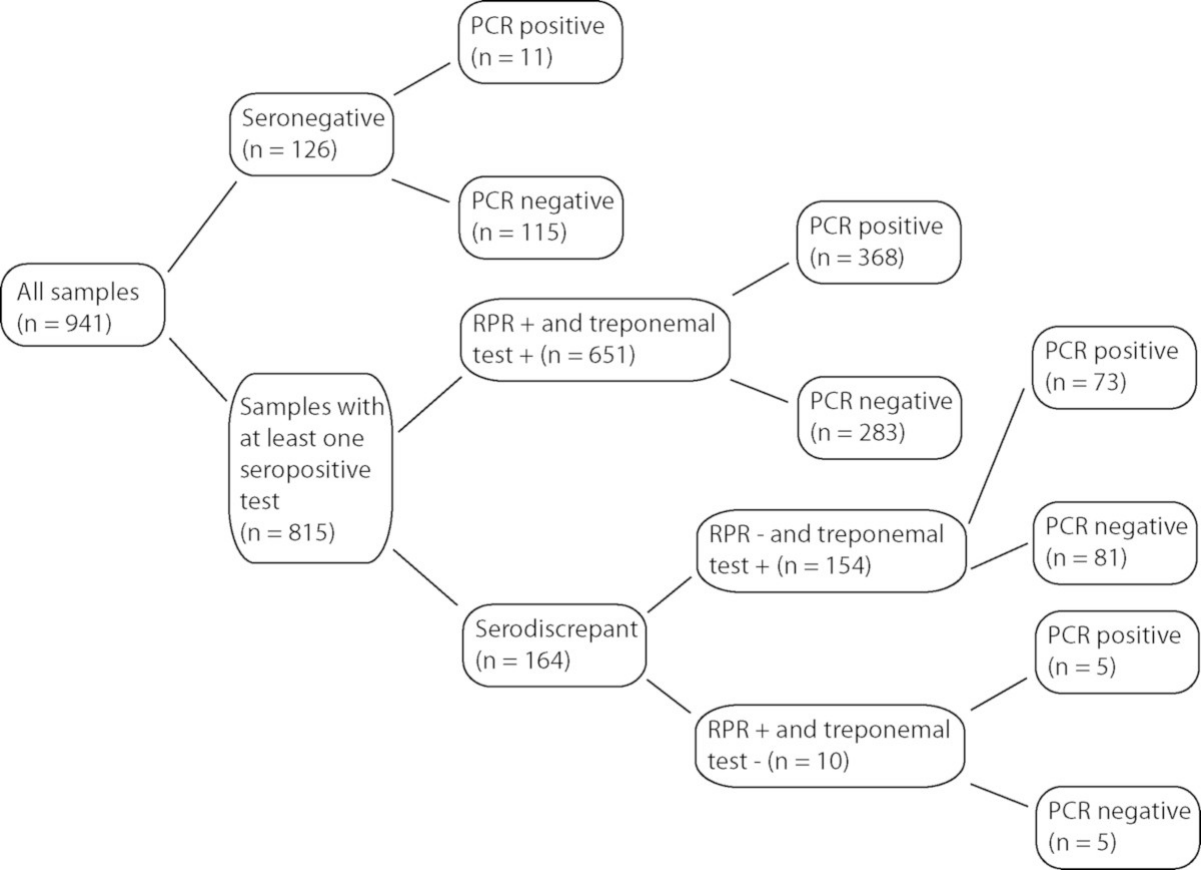
Overview of serology and PCR results. PCR positive samples were found among seronegative samples (8.7%), seropositive samples (56.5%), and serodiscrepant samples (47.6%).

An overview of serological and PCR results is shown in Fig 1. Among the 941 samples, 126 were seronegative (i.e., negative for both the non-treponemal and treponemal tests), 651 were syphilis-seropositive (positive for both non-treponemal and treponemal tests), and 164 were serodiscrepant (positive in one type of serological test only; i.e., either non-treponemal or treponemal). Among the seronegative samples, there were 11 PCR (8.7%) positive samples. Among the seropositive samples, 368 (56.5%) were PCR positive and 283 (43.5%) were PCR negative. Most of serodiscrepant samples (n = 154) were RPR-negative and treponemal test-positive, of which 73 were PCR positive (47.4%) and 81 were PCR negative (52.6%). Of the 10 RPR-positive and treponemal test-negative samples, 5 were PCR positive (50.0%) and 5 were PCR negative (50.0%). Altogether, 89 samples were PCR positive and seronegative or PCR positive and serodiscrepant. Whole blood and swab samples were taken at the same time from 108 patients. The PCR results of the patients´ samples are shown in Table 1. The PCR positivity was 64.8% for swabs and 50.9% for whole blood samples.

**Table 1:** PCR results of 108 patients with parallel whole blood and swab sample.

	Whole blood PCR positive	Whole blood PCR negative	Total no. of samples
**Swab PCR positive**	47	23	70
**Swab PCR negative**	8	30	38
**Total no. of samples**	55	53	108

### Agreement between serology and PCR tests, sensitivity, specificity, and predictive values

Agreement between serology and PCR tests was determined for the entire sample set as well as for a subsample set without serodiscrepant results. For the complete set of 941 samples, agreement was 59.6%, and the kappa value was 0.209 (95% CI 0.168–0.250) indicating *slight* agreement. For the subsample set, the agreement was 62.2% and the kappa value was 0.256 (95% CI interval 0.207–0.304) indicating *fair* agreement of both tests.

Sensitivity, specificity, and predictive values were determined for serology and PCR according our syphilis case definition. For serology, sensitivity was 89.3% (95% CI 86.86–91.29%), specificity was 100% (95% CI 98.18–100%), positive and negative predictive values were 100% and 69.3% (95% CI 64.98–73.32%). For PCR, sensitivity was 72.3% (95% CI 69.48–75.06%), specificity was 100% (95% CI 98.18–100%), and positive and negative predictive values were 100% and 41.5% (95% CI 39.14–43.95%).

### Analysis of samples from seropositive and serodiscrepant patients

Detailed characteristics of syphilis-seropositive (n = 651) and serodiscrepant (n = 164) samples are shown in S2 Table. This sample set contained 411 swab samples (50.4%) and 404 whole blood samples (49.6%). Swab samples were PCR-positive more often (n = 314; 76.4%) than whole blood samples (n = 132; 32.7%; p = 0.0001).

We compared PCR-positivity with positivity for the TPPA test, the test for IgM, the test for IgG, and RPR individually among different sample sources (i.e., swabs vs. whole blood). RPR log titer was then compared between PCR positive and PCR negative samples for one sample source.

Among 411 swab samples, an association between PCR-positivity and positive results of treponemal serological tests was found, including TPPA (p = 0.0001), ELISA/WB IgM (p = 0.0001), and ELISA/WB IgG (p = 0.0011). Although the average RPR log titer was higher in PCR-positive samples, no significant association was found between PCR-positivity and RPR-positivity/negativity or the average log value of RPR.

An association between PCR-positivity in whole blood samples and treponema specific serological tests was found for ELISA/WB IgM (p = 0.0001). Moreover, in samples that were positive for IgG and negative for IgM (n = 103), whole blood samples were more often PCR negative (p = 0.0001). The average titer log value was higher in PCR-positive blood samples (p = 0.0406) compared to PCR-negative samples.

### Analysis of samples with negative serology and samples with discrepant serological results

Altogether, 280 RPR negative samples were analyzed in this study. These samples included RPR-negative and treponemal test-negative samples (n = 126; 45%) and samples with discrepant serology (n = 154; 55%). A total of 11 samples were PCR-positive in patients with negative serology, while 73 samples were both PCR-positive and treponemal test-positive.

There were also RPR positive samples that were treponemal test-negative (n = 10). Of these, five samples were PCR positive, i.e. three swabs (out of 5) and two blood samples (out of 5).

### Comparison of PCR-positivity in different syphilis stages

PCR positivity in swab samples and in whole blood samples differed in patients in different stages of the disease. An overview of PCR detection rates is shown in Table 2. The PCR detection rate was highest in samples from patients with primary syphilis. The lowest PCR-positivity rates were found in patients with an undetermined syphilis stage, which was statistically different from both the primary (swabs, p = 0.0005; whole bloods, p = 0.0065) and secondary stage (whole bloods, p = 0.0033). In all stages, PCR-positivity of whole blood samples were lower compared to swabs (primary stage, p = 0.0001; secondary stage, p = 0.0001; undetermined stage, p = 0.0001).

**Table 2:** PCR detection rates in seropositive samples taken from patients in different stages of syphilis.

Syphilis stage (total no. of seropositive samples)	Swab PCR positivity rate (total no. of positive samples/ total no. of samples)	Whole blood PCR positivity rate (total no. of positive samples/ total no. of samples)
Primary stage (n = 292)	84.3% (166/197)	42.1% (40/95)
Secondary stage (n = 148)	75.9% (41/54)	41.5% (39/94)
Undetermined stage (n = 211)	64.1% (50/78)	21.1% (28/133)
Total (n = 651)	78.1% (257/329)	33.2% (107/322)

## Discussion

In this study, a suspected syphilis infection was defined as having clinical signs consistent with a primary or secondary infection and/or relevant anamnestic data. However, in several cases, the stage was not determined. This group of samples either comprised cases with missing data or came from patients that were clinically asymptomatic and were found to be seropositive during routine/preventive screening. The number of these samples (n = 306) was less than one-third of the samples analyzed in this study.

Sensitivity, specificity, positive and negative predictive values, were 72.3%, 100%, 100%, 41.5% for PCR and 89.3%, 100%, 100%, 69.3% for serology, respectively. These results are comparable with the results reported by Noda et al. [15] with slightly lower sensitivity and negative predictive value of PCR and slightly higher sensitivity and negative predictive value of serology. However, Noda et al. [15] only analyzed samples taken from patients in the primary stage. Higher PCR sensitivity was reported by Leslie et al. [26] and Palmer et al. [27]; however, this was likely a result of excluding blood samples of those with undetermined syphilis stages. The sensitivity and specificity of serology found in our study correlate well with serological test values reported by Larsen et al. [9].

Approximately half of our samples were swabs, and the other half were whole blood samples. As reported in other studies [7, 8, 28], swab samples yielded positive PCR results more frequently than whole blood samples. The positivity of swab samples was 76.4%, which is a noteworthy detection rate, which could, in the majority of cases, confirm the diagnosis by directly detecting the causative agent. In contrast, the detection rate in blood (32.7%) was considerably lower. This was partly a result of the lower number of treponemes in the samples [29] and partly because of the fact that the majority of samples from those with an undetermined syphilis stage were also whole blood PCR negative samples. These samples may, in part, represent clinically asymptomatic patients with non-active syphilis resulting from previous intentional syphilis treatment or unintentional syphilis treatment linked to treatment for a different indication. Similar results related to PCR positivity of swabs (77.8%) and whole blood (34.8%) were also obtained when using a subset of samples from patients with both positive non-specific and specific treponemal tests were analyzed. Interestingly, when seropositive patients in the primary and secondary stage (n = 440) were analyzed, the PCR detection rate in swab and whole blood increased to 82.5% and 41.8%, respectively, suggesting that clinically asymptomatic seropositive patients likely represent patients with a history of a previous syphilis infection rather than a current infection. An overview of PCR positivity based on syphilis serology and syphilis stage is shown in Table 3.

**Table 3:** PCR detection rates in swab and whole blood samples relative to syphilis serology and syphilis stage.

Samples (total no. of samples)	Swab PCR positivity rate (total no. of positive samples; total no. of samples)	Whole blood PCR positivity rate (total no. of positive samples; total no. of samples)
All seropositive and serodiscrepant samples (n = 815)	76.4% (314/411)	32.7% (132/404)
RPR and treponemal test-positive samples (n = 651)	77.8% (256/329)	34.8% (112/322)
RPR and treponemal test-positive samples from primary and secondary stage (n = 440)	82.5% (207/251)	41.8% (79/189)
RPR and treponemal test-positive samples from primary stage (n = 292)	84.3% (166/197)	42.1% (40/95)

The highest PCR detection rate was found in RPR-positive and treponemal test-positive samples from patients in the primary stage; the rate was 84.3% for swabs and 42.1% for whole blood samples. The agreement between serology and nested PCR in patients in the primary stage was 84.3% in for swab samples, which is quite high, although it was lower than the 95% described by Leslie et al. [26]. However, Leslie et al. [26] analyzed a limited number of samples collected from only 55 patients. In Table 4, the PCR detection rates in different serological profiles in primary stage are shown.

**Table 4:** PCR positivity rates in different serological profiles in primary syphilis.

Serological profile	Total no. of samples	PCR positivity rate (no. of positive samples)
Seropositive	292	70.5% (n = 206)
RPR-negative, treponemal test-positive	60	75% (n = 45)
RPR-positive, treponemal test-negative	6	66.7% (n = 4)
Seronegative	6	66.7% (n = 4)

Interestingly, detection rates for swabs and whole blood converged in samples from patients with two available samples taken at the same time, swabs 64.8% and whole blood 50.9%. This trend increased further when the samples came from those in the primary and secondary stage, swabs 76.5%, and whole blood 61.7%. All these findings indicate that (1) the PCR detection rate in swab samples is considerably higher compared to whole blood samples and (2) PCR detection in whole blood samples of patients with primary and secondary syphilis is over 40%. Since syphilis detection occurs primarily during the primary and secondary stages in most countries, nested PCR detection could make a valuable contribution to syphilis diagnostics.

One hundred twenty-six samples (13.4%) analyzed in this study were seronegative for both treponemal and non-treponemal serological tests. Of these samples, almost 9% (n = 11) were found to be PCR positive, indicating that PCR can detect treponemes in the very early stages of infection when patients may be seronegative. Almost all these samples were genitoanal swabs (10 out of 11) and just one of the samples was a whole blood sample. These samples were most likely taken from patients within “the seronegative window” of syphilis infection, therefore nested PCR detection could be of considerable help in making a diagnosis in the early stages of a syphilis infection. PCR positive samples from seronegative patients have been identified by several authors [13–15, 26, 27, 30]; PCR detection in these patients thus offers a definitive syphilis diagnosis. The other samples that were seronegative (n = 115) were also PCR negative and thus likely represent patients with symptoms mimicking primary syphilis, although not actually infected with syphilis treponemes.

Approximately 16% of all samples (n = 164) had discrepant results relative to non-treponemal and treponemal serology tests. While the majority of samples were RPR-negative and positive for the treponemal test, only 10 samples (6.1%) were RPR-positive and negative for the treponemal test. This finding supports the superiority of the treponemal test in the identification of syphilis. In fact, as shown in this and other studies [31, 32], PCR positivity has been found to be positively associated with the results of the treponemal serological test. The group of RPR-negative and treponemal test-positive samples could, at least in part, represent samples from patients with a previous syphilis infection, especially in PCR negative samples taken from patients with an undetermined stage (n = 68). On the other hand, the PCR positivity in a group of RPR-negative and treponemal test-positive samples showed an active infection as treponemal tests are positive sooner during infection compared to RPR.

In an additional 10 samples, the results of serology showed RPR-positivity with negative results for the treponemal test. Out of these samples, 50% were PCR negative, and 50% PCR positive. The RPR reaction becomes positive in the later stages of syphilis compared to the IgG and IgM response, suggesting that PCR negative patients were likely seronegative and tested RPR-positive for other reasons than TPA infection, e.g., other pathogenic treponemal infections, acute bacterial or viral infections, or vaccination. PCR positive samples are therefore likely again taken from patients in “the seronegative window” of a syphilis infection. Together with seronegative patients, nested PCR detected 16 cases of very early syphilis, which represented 3.5% of all PCR positive samples. Although this contribution is relatively limited in comparison to serological testing, it could considerably limit the transmission of syphilis by patients who were falsely reassured that they did not have syphilis.

A statistically significant association between PCR-positivity and positive results from treponemal serological tests but not with RPR was found for swab samples, indicating that direct (PCR) and indirect TPA detection (IgG, IgM) were correlated and differed from non-treponemal tests, such as the RPR. Similar findings came from an analysis of whole blood samples in which PCR positivity was correlated with the presence of IgM, while the absence of IgM combined with the presence of IgG was correlated with PCR negativity. Our findings are in agreement with those reported by Casal et al. [31], i.e., there was an association between PCR and tests for IgM. In addition, a better correlation between PCR and treponemal test vs. non-treponemal VDRL was reported by Shuel et al. [32]. PCR positivity of whole blood samples appears to correlate with the early stages of syphilis. PCR detection of treponemes in whole blood gets increasingly more difficult the older the syphilis infection. These findings are consistent with the results of PCR being dependent on the number of treponemes present in whole blood samples.

Taken together, and despite the limitations of PCR detection of syphilis treponemes in clinical samples, this study shows that nested PCR can improve syphilis diagnostics, especially in seronegative patients and in patients with discrepant serology.

## Supporting information

S1 Table PCR detection. (PDF)

S2 Table Basic characteristics of patients with seropositive and serodiscrepant samples. (PDF)
