# Supplementary material for: A retrospective study on nested PCR detection of syphilis treponemes in clinical samples: PCR detection contributes to the diagnosis of syphilis in patients with seronegative and serodiscrepant results
Source: PLoS One. 2020 Aug 20;15(8):e0237949. doi: 10.1371/journal.pone.0237949 (PMC7446855; doi:10.1371/journal.pone.0237949)
Supplement: S1 Table — (PDF) [file pone.0237949.s001.pdf]

S1 Table
A. List of primers

Locus	External primers (5-3)	Length of PCR product	Internal primers (5-3)	Length of PCR product
**TP0136**	AACCCGTTAGCGCCCAACAT	1789 bp	AGTGTCTTCCTCGTCCGTTC	1206 bp
	TCCCAGCTCAGCCGAATCTC		CACGTGGTGGTGTCAAACTT	
**TP0548**	TGGGGCACTAAACCGGAAGA	1567 bp	GCGGTCCCTATGATATCGTGT	1065 bp
	TACGGGCATTTGCGGATAGG		GAGCCACTTCAGCCCTACTG	
**TP0705**	GGTCTATATGCAGCCCTTCTTC	1181 bp	TGCGGCTTATCCTGATGAATAG	803 bp
	GCTTGAGAACGATACCGGATAC		TATTCTGCGGCGTTGGATAG	
**23S rDNA**[1]	CGAAGGGAAGCAGGTGTAGT	1666 and 1658 bp	GTACCGCAAACCGACACAG	629 bp
	GCGCGAACACCTCTTTTTAC		AGTCAAACCGCCCACCTAC	
	GAACCGTCCCTGAAAACTCA			
**TP0319**	CTGCTCATCGGCTGCTCTA	733 bp	GAAGGTGGTGACTTCGTCGT	451 bp
	ACCACAGACTTCGACCCATC		CAAAACCCGCTTCAAAGAGA	
**TP0105**	TTCTGTGCTCACGTCTGGTC	637 bp	TGCGCGTGTGCGAATGGTGTGGTC	376 bp
	TGCAACCATCGTATCGAAAA		CACAGTGCTCAAAAACGCCTGCACG	

[1]Both copies of 23S rDNA gene were amplified

B. First step reaction with 1 µl of DNA

Compound	Volume (µl)	Final volume
water	16.3	25 µl
2.5 mM deoxynucleotide triphosphate (dNTP)	2	
5x PS GXL buffer	5	
Primer F (100 pmol/µl)	0.095	
Primer R (100 pmol/µl)	0.095	
DNA**	1	
PrimeSTAR GXL polymerase*	0.5	

*Takara, BioEurope, France
** DNA of TPA strain Nichols (5 pg/µl) was used as a positive control; distilled water was used as a negative control.

Phase	Step	Temperature	Time	No. of cycles
1	First denaturation	94 °C	1 min	1
2	Denaturation	98 °C	10 s	8
	Annealing	68 °C*	15 s	
	Extension	68 °C	1 min 45 s	
3	Denaturation	98 °C	10 s	35
	Annealing	61 °C	15 s	
	Extenaion	68 °C	1 min 45 s	
4	Final extension	68 °C	7 min	1

*−1.0 °C per cycle

C. First step reaction with 10 µl of DNA

Compound	Volume (µl)	Final volume
water	11.5	25 µl
10 mM dNTP mixture	0.5	
ThermoPol Reaction buffer	2.5	
Primer F (100 pmol/µl)	0.25	
Primer R (100 pmol/µl)	0.25	
DNA**	10	
Taq polymerase*	0.1	

*5,000 U/ml; New England BioLabs, Ipswich, MA
** DNA of TPA strain Nichols (5 pg/µl) was used as a positive control; distilled water was used as a negative control.

Phase	Step	Temperature	Time	No. of cycles
1	First denaturation	94 °C	1 min	1
2	Denaturation	94 °C	20 s	8
	Annealing	55 °C*	20 s	
	Extension	72 °C	1 min 45 s	
3	Denaturation	94 °C	20 s	35
	Annealing	48 °C	20 s	
	Extension	72 °C	1 min 45 s	
4	Final extension	72 °C	7 min	1

* −1.0 °C per cycle

Note: Conditions of PCR amplification of TP0319 and TP0105: 94 °C (1 min); 94 °C (30 s), 58 °C (30 s), 72 °C (1 min) for 30 cycles; and 72 °C (10 min).

D. Second step reaction

Compound	Volume (µl)	Final volume
water	20.5	25 µl
10 mM dNTP mixture	0.5	
ThermoPol Reaction buffer	2.5	
Primer F (100 pmol/µl)	0.25	
Primer R (100 pmol/µl)	0.25	
PCR product from first step	1	
Taq polymerase*	0.1	

*5,000 U/ml; New England BioLabs, Ipswich, MA

Phase	Step	Temperature	Time	No. of cycles
1	First denaturation	94 °C	1 min	1
2	Denaturation	94 °C	30 s	40
	Annealing	48 °C	30 s	
	Extension	72 °C	1 min 15 s	
3	Final extension	72 °C	7 min	1

Note: Conditions for TP0319 and TP0105: 94 °C (1 min); 94 °C (30 s), 58 °C (30 s), 72 °C (1 min) for 40 cycles; and 72 °C (10 min).
