# Supplementary material for: A retrospective study on nested PCR detection of syphilis treponemes in clinical samples: PCR detection contributes to the diagnosis of syphilis in patients with seronegative and serodiscrepant results
Source: PLoS One. 2020 Aug 20;15(8):e0237949. doi: 10.1371/journal.pone.0237949 (PMC7446855; doi:10.1371/journal.pone.0237949)
Supplement: S2 Table — (PDF) [file pone.0237949.s002.pdf]

S2 Table. Basic characteristics of patients with seropositive and serodiscrepant samples

Clinical characteristics			Swab samples (n = 411)		Whole-blood samples (n = 404)	
			PCR positive (314; 257 SP, 57 SD)	PCR negative (97; 72 SP, 25 SD)	PCR positive (132; 112 SP, 20 SD)	PCR negative (272; 210 SP, 62 SD)
**Mean age**	Men	All	35.7 (0 – 71)	34.7 (16 – 71)	35.4 (0 – 71)	36.6. (16 – 77)
		SP	36 (0 – 71)	35.1 (16 – 71)	35.9 (0 – 71)	36.3 (16 – 67)
		SD	34.4 (17 – 61)	33.2 (23 – 46)	32.5 (18 – 43)	37.5 (20 – 77)
	Women	All	27.5 (0 – 53)	32 (18 – 61)	31.3. (20 – 56)	31.9 (0 – 73)
		SP	27.1 (0 – 53)	26.4 (18 – 30)	30.1 (20 – 56)	29.7 (0 – 53)
		SD	30.4. (20 – 38)	36.5. (20 – 61)	51 (51)*	35.8 (21 – 73)
**Sex, n (%)**	Men	All	275 (87.6)	79 (81.4)	114 (86.4)	223 (82)
		SP	223 (86.8)	64 (88.9)	95 (84.8)	178 (84.8)
		SD	52 (91.2)	15 (60)	19 (95)	45 (72. 6)
	Women	All	39 (12.4)	18 (18.6)	18 (13.6)	48 (17.6)**
		SP	34 (13.2)	8 (11.1)	17 (15.2)	31 (14.8)***
		SD	5 (8.8)	10 (40)	1 (5)	17 (27.4)
**Serology**	TPPA/ TPHA	All	291 P, 6 N, 17 n.d.	80 P, 13 N, 4 n.d.	117 P, 3 N, 12 n.d.	252 P, 10 N, 10 n.d.
		SP	245 P, 5 N, 12 n.d.	67 P, 2 N, 3 n.d.	112 P, 10 n.d.	202 P, 1 N. 7 n.d.
		SD	46 P, 1 N, 5 n.d.	13 P, 11N, 1 n.d.	15 P, 3 N. 2 n.d.	50 P, 9 N, 3 n.d.
	RPR	All	259 P, 55 N average log-3.26	72 P, 23 N, 2 n.d. average log: -2.77	112 P, 18 N, 2 n.d. average log: -3.63	210 P, 58 N, 4 n.d. average log: -3.08
		SP	257 P, 5 N average log: -3.25	70 P, 2 n.d. average log: -3.78	110 P, 2 n.d. average log: -4.25	206 P, 4 n.d. average log: -3.99
		SD	2 P, 50 N average log: -0.07	2 P, 23 N average log: -0.08	2 P, 18 N average log: -0.30	4 P, 58 N average log: -0.05
	IgM	All	285 P, 22 N, 6 n.d.	71 P. 23 N, 3 n.d.	116 P, 11 N, 5 n.d.	195 P, 66 N, 11 n.d.
		SP	246 P, 12 N, 3 n.d.	59 P, 10 N, 3 n.d.	104 P, 4 N, 4 n.d.	189 P, 24 N, 10 n.d.
		SD	39 P, 10 N, 3 n.d.	12 P, 13N	12 P, 7 N, 1 n.d.	6 P, 42 N, 1 n.d.
	IgG	All	301 P, 5 N, 8 n.d.	85 P, 9 N, 3 n.d.	116 P, 4 N, 12 n.d.	245 P, 15 N, 12 n.d.
		SP	256 P, 2 N, 4 n.d.	69 P, 3 n.d.	104 P, 8 n.d.	197 P, 2 N, 11 n.d.
		SD	45 P, 3 N, 4 n.d.	16 P, 9 N	12 P, 4 N, 4 n.d.	48 P, 13 N, 1 n.d.
**Syphilis stage**	primary	All	203	41	52	62
		SP	166	31	40	55
		SD	37	10	12	7
	secondary	All	42	13	39	57
		SP	41	13	39	55
		SD	1	0	0	2
	others	All	69	43	41	153

	SP	50	28	33	100
	SD	19	15	8	53

SP – seropositive, SD - serodiscrepant
P – positive, N – negative, n.d. – not determined
* one woman in age of 51
** unknown sex: 1 (0.4%)
*** unkown sex: 1 (0.4%)
